# Impact of comorbid borderline personality disorder on the outcome of inpatient treatment for anorexia nervosa: a retrospective chart review

**DOI:** 10.1186/s40479-021-00149-7

**Published:** 2021-03-11

**Authors:** Ulrich Voderholzer, Matthias Favreau, Sandra Schlegl, Johannes Baltasar Hessler-Kaufmann

**Affiliations:** 1 Schoen Clinic Roseneck, Prien am Chiemsee, Am Roseneck 6, D-83209 Prien am Chiemsee, Germany; 2 Department of Psychiatry and Psychotherapy, University Hospital of Freiburg, Freiburg, Germany; 3 Department of Psychiatry and Psychotherapy, University Hospital, LMU Munich, Munich, Germany

**Keywords:** Anorexia nervosa, Borderline personality disorder, Eating disorder, Inpatient treatment, Routine care, Multilevel modeling

## Abstract

**Background:**

Data on patients with anorexia nervosa (AN) and comorbid Borderline personality disorder (AN+BPD) are scarce. Therefore, we investigated (1) whether patients with AN and AN+BPD differ in characteristics related to admission to, discharge from, and course of specialized inpatient eating disorder treatment and (2) how comorbid BPD affects treatment outcome.

**Method:**

One-thousand one-hundred and sixty inpatients with AN (97.2% female, 5.9% with comorbid BPD; mean age = 26.15, *SD* = 9.41) were administered the Brief Symptom Inventory (BSI), the Eating Disorder Inventory 2 (EDI-2), and the Global Assessment of Functioning (GAF) at admission and discharge. Data were extracted by a retrospective chart review of naturalistic treatment data. Age, sex, weekly weight gain, length of stay, and discharge characteristics were compared with independent *t*-tests and χ^2^-tests. Changes in outcome variables, including body mass index (BMI), were analyzed with longitudinal multilevel mixed-effects models.

**Results:**

No differences in age or sex were found between patients with AN and AN+BPD, but groups differed in previous inpatient treatments, BMI at admission, and frequency of at least one additional comorbidity with higher values for AN+BPD. Higher levels of disorder-specific and general psychopathology at admission were found for AN+BPD. Patients with AN showed statistically significant improvement in all examined variables, patients with AN+BPD improved in all variables except EDI-2 body dissatisfaction. Strongest improvements in patients with AN+BPD occurred in BMI (Cohen’s *d* = 1.08), EDI-2 total score (Cohen’s *d* = 0.99), EDI-2 interpersonal distrust (*d* = 0.84). Significant Group x Time Interactions were observed for BSI GSI, GAF, and EDI-2 body dissatisfaction, indicating a reduced benefit from inpatient treatment in AN+BPD. At discharge, no differences were found in weekly weight gain, BMI, length of stay, or discharge characteristics (e.g., ability to work, reason for discharge), however, patients with AN+BPD were more frequently treated with medication.

**Conclusions:**

Patients with AN+BPD differ from patients with AN in that they show higher general and specific eating disorder psychopathology and only partially improve under specialized inpatient treatment. In particular, aspects of emotion regulation and core AN symptoms like body dissatisfaction and perfectionism need to be even more targeted in comorbid patients.

**Supplementary Information:**

The online version contains supplementary material available at 10.1186/s40479-021-00149-7.

## Background

In anorexia nervosa (AN), psychiatric comorbidities are the rule rather than the exception. With rates of around 50%, personality disorders (PDs) are among the most prevalent co-occurring conditions [1]. Next to avoidant, dependent, and obsessive-compulsive PDs, Borderline personality disorder (BPD) seems to be especially prominent in AN, with roughly every fifth patient being affected [2]. High rates of comorbidity might not be surprising, as both AN and BPD potentially share core features like affective instability or deficits in emotion regulation. So far, studies on the clinical presentation of persons with AN + BPD and their response to treatment are lacking.

Comorbid PDs are generally thought to complicate the treatment of eating disorders and lead to a worse prognosis [3–6]. No moderation effects of personality disorders on treatment outcome were found in a systematic review for binge-eating disorder (BED), while comorbid personality disorders predicted a worse treatment response in bulimia nervosa (BN) [3]. None of the included studies with AN examined personality disorders in general as a predictor, moderator, or mediator on treatment outcome [3]. In a mixed sample, patients with comorbid PD scored higher than patients without PD on EDI subscales Drive for Thinness, Body dissatisfaction, Ineffectiveness, perfectionism, interpersonal distrust, and interoceptive awareness at initial assessment and a 5-year follow-up, but no significant Group x Time Interactions could be observed [4]. Patients with comorbid PDs also showed a more severe general psychopathology (anxiety, depression, somatization, phobic anxiety, paranoid ideation, psychoticism, global severity scales) [4]. However, patients with comorbid PDs did not differ in symptomatic changer over time from patients without PDs [4]. A meta-analysis of predictors of treatment outcome across all eating disorders revealed a small correlation (r = .012) between the absence of personality disorders and better treatment outcomes [5].

Overall, these findings suggest that a comorbid PD is related to more severe psychopathology and reduced treatment response. This may be the case because of more generalized dysfunctions in psychological processing and functioning across most areas of individuals’ everyday life, which are stable over time and across different situations. According to DSM-5, personality disorders are enduring, inflexible, and maladaptive patterns of internal experiences and behaviors. Besides, personality features are often more ego-syntonic and not perceived as problematic by the affected individuals by themselves. Thus, in conclusion, eating disorder symptomatology, often based on deficits of fundamental psychological functions like impulse regulation or interoceptive awareness, can be more difficult to treat in individuals with comorbid personality disorders due to more persistent and widespread dysfunctions across the life span. However, how and to what extent specific PDs contributed to reduced treatment responses can hardly be quantified without a distinction between individual PDs.

Evidence regarding the impact of specific PDs like BPD on treatment outcome in eating disorders is scarce. In a mixed sample of 19 patients with eating disorders, higher severity of borderline psychopathology at baseline was associated with reduced levels of global functioning, and more severe eating disorder symptoms [15]. Also, baseline borderline symptom severity was negative correlated with life satisfaction and change in eating disorder symptoms at a 3-year follow-up [15]. A 5-year follow-up study in another mixed sample of 30 patients revealed that only BPD served as a significant predictor of treatment outcome in EDI scales [4]. Furthermore, Zanarini et al. [2] reported high rates (> 70%) of diagnostic migration to other eating disorders during a 10-year follow-up for individuals with BPD and comorbid AN or BN. Nevertheless, lower recurrence rates were recorded for AN and BN compared to eating disorders not otherwise specified (EDNOS) among patients who achieved remission from their eating disorder [2]. In BN, a comorbid BPD was found to complicate treatment with regard to a worse presentation at the beginning of treatment, longer stays, higher dropout rates, and less improvement [7–11]. Preliminary findings confirmed these associations in samples with AN and comorbid PDs in general and yielded higher dropout rates, lower mean age of onset for AN, lower lifetime minimum BMI, more hospitalizations due to AN for comorbid patients, tendencies toward chronicity, and a higher mean weight, but no differences in total weight gain during treatment [12–14].

Studies with small and heterogeneous samples only indicated a similarly poor response to treatment across different therapeutic approaches (Dialectical Behavior Therapy, Cognitive Behavior Therapy), settings (inpatient, outpatient), populations (children and adolescents with subsequent personality disorder diagnosis in adulthood compared to adults) and study lengths [7, 15, 16] in patients with AN and BPD (AN+BPD) as with any comorbid PD.

To sum up, previous studies were mainly based on mixed samples, investigated the impact of PDs in general, or compared treatment outcomes in comorbid patients between different eating disorders (e.g., AN+BPD vs. BN + BPD). Results revealed a reduced treatment response for patients with eating disorders and comorbid PDs. However, these studies failed to investigate how specific domains are differentially affected by individual comorbid PDs like BPD and could account for reported reduced treatment response in patients with eating disorders. Such differences in specified aspects of disorder-specific and general psychopathology between patients with and without comorbid PDs have hardly been investigated to date, especially in AN. Consequently, details of these associations between PDs and treatment outcome, as, for example, the differential influence of comorbid BPD on individual facets of eating disorders symptoms in AN, remains unclear. For example, reduced weight gain in patients with AN+BPD compared to AN could be attributed to differences in a number of domains, e.g., body dissatisfaction, dysfunctional emotion regulation, or interoceptive awareness.

Knowledge about the additional and aggravated impairment in specific domains associated with a comorbid BPD may help to identify and specifically address special treatment needs of comorbid patients and, thereby, improve treatment for a large group of patients that suffers from multiple severe mental disorders and does not yet receive adequate help because standardized disorder-specific, symptom-centered treatments may do not fully match the specials needs of this group. It seems possible that a more individualized, comprehensive treatment option is needed for comorbid patients with AN+BPD to achieve similar treatment effects as in patients with AN.

Our study aimed to compare large samples of inpatients with AN and AN+BPD in (1) characteristics at admission and discharge and (2) response to treatment concerning weight, core eating disorder symptoms, depressive symptoms, general psychopathology, and psychosocial functioning. Based on our clinical expertise and previous findings in the literature, we hypothesized that

1. patients with AN and comorbid BPD show higher levels of eating disorder as well as general psychopathology at admission and discharge and
2. a comorbid BPD is associated with reduced inpatient treatment response in general and
3. specific facets related to both AN and BPD (e.g., impulse regulation, interpersonal distrust, the general level of psychosocial functioning) are differentially affected by inpatient treatment depending on comorbid BPD. For this purpose, we mainly focus on Group x Time Interactions in selected outcome parameters.

## Method

### Study sample

The sample consisted of consecutive patients admitted to the Schoen Clinic Roseneck in Prien, Germany, for inpatient treatment of AN between January 01, 2013, and December 31, 2017.

The Schoen Clinic is highly specialized in the treatment of adolescents and adults with AN and other eating disorders. Offered treatment is addressing both physical weight gain as well as psychological issues like dietary changes, body exposure, body acceptance, or cognitive restructuring [17, 18]. We included patients with AN (F50.0, F50.1), who had a BMI ≤ 17.5 and were 18 years or older. Clinical Diagnoses of AN and BPD (F63.3) according to ICD-10 were given by the treating therapists, who were experienced clinicians in psychotherapy or therapists in training under supervision of experienced clinicians. All patients signed informed consent to the use of their routine data for scientific purposes. Data were assessed by retrospective chart review. There were no restrictions made regarding further comorbidities. Structured diagnostic assessment for eating disorders included clinical interviews as well as various clinician-rated and self-report questionnaires (e.g., Eating Disorder Inventory 2 (EDI-2), Munich ED-Quest, Eating Disorder Examination-Questionnaire (EDE-Q), Compulsive Exercise Test (CET), Compulsive Exercise Scale (CES), Brief Symptom Inventory (BSI), Beck Depression Inventory-II (BDI-II), Patient Health Questionnaire-9 (PHQ-9), Brief Resilience Scale (BRS) or Satisfaction with Life Scale (SWLS)). Among other measures, the BSI yields a Global Severity Index (GSI), which aggregates all available information and gives a general estimate of psychopathology severity.

All procedures were in accordance with the ethical standards of the institutional review board of the LMU Munich and with the 1964 Helsinki declaration and its later amendments. According to the guidelines by the institutional review board of the LMU Munich, retrospective studies conducted on already available, anonymized data are exempt from requiring ethics approval.

### Materials and procedure

Assessment procedures included a range of psychometric questionnaires for all patients both at admission and discharge. The Eating Disorder Inventory 2 (EDI-2 [19, 20]) assesses general eating disorder pathology and yields a total mean score and eleven mean subscores that cover typical eating disorder psychopathology. The subscores pertain to the following scales: drive for thinness, bulimia, body dissatisfaction, ineffectiveness, perfectionism, interpersonal distrust, interoceptive awareness, maturity fears, asceticism, impulse regulation, and social insecurity. The items are rated on a 6-point Likert-scale ranging from “always” to “never”. The validation of the German version of the EDI-2 in a sample of patients with AN and BN revealed acceptable to excellent internal consistency with Cronbach’s α = .73–.93 for the different subscales and retest reliability between r = .81 and r = .89 [20].

Patients also completed the Brief Symptom Inventory (BSI, [21, 22]). Among other measures, the BSI yields a score for Depression and a Global Severity Index (GSI), which summarizes all information and gives a general estimate of the degree of psychopathology. Studies on validation yielded satisfying internal consistency for the subscale Depression with Cronbach’s α = .72 to .85 and α = .92–.96 for the Global Severity Index GSI [22].

All patients were rated by their therapists on the Global Assessment of Functioning (GAF, described in the DSM-IV [23]), which measures a person’s psychosocial and occupational functioning with scores ranging from 0 to 100. Higher scores indicate better functioning.

### Inpatient treatment

According to the current German national guideline “Diagnosis and treatment of eating disorders” (see Supplement 1), inpatient treatment is focused on physical stabilization and should foster transition into day-clinic or outpatient care to achieve full recovery. The main treatment goals are normalization of body weight and changing eating behavior and attitudes through multimodal psychotherapy. For complete recovery (e.g., stable and regular weight, sufficient quality of life, abstinence from binge-purging behaviors, normalized body image), a subsequent outpatient treatment in local services is usually needed.

Patients received intensive multimodal treatment comprising individual psychotherapy (1 or 2 sessions of 50 min per week) and an eating-disorder specific manualized group therapy, both based on cognitive behavioral therapy. Individual psychotherapy was not manualized but addressed disorder-specific issues such as psycho-education about eating disorders, personalized case formulation, dietary changes, body exposure, body acceptance, cognitive restructuring, and relapse prevention to maintain achieved weight gain and prepare patients for the transition into outpatient care before discharge. Patients were obliged to eat three meals and two snacks with a total amount of 2000–2100 kcal per day to achieve sufficient weight gain and received mealtime support and supervision but were in charge of their eating and related compensatory behavior themselves. At all times, they had free access to a cafeteria, supermarket, and lavatories.

Patients were treated by a multidisciplinary team consisting of physicians, trained psychotherapists or clinical psychologists, co-therapists from the nursing-staff, and dieticians. Patients were medically monitored by physicians to control for potential laboratory abnormalities and other somatic complications. Patients’ safety during weight gain diet was ensured through medical monitoring (e.g., for laboratory abnormalities) by physicians. Other groups included art therapy, sports therapy, cooking training, and social skills training. A skills-group based on principles of Dialectic Behavioral Therapy served as the only specific additional treatment component for BPD. Individual and group psychotherapies were conducted by trained clinical psychologists and psychiatrists, who received regular supervision from experienced therapists. Next to treating psychotherapists and physicians, patients had individual co-therapists from the nursing staff, who were available for crisis intervention, individual therapy sessions, and representing general contact persons on the wards.

### Statistical analysis

Differences between diagnostic groups were analyzed with independent samples *t*-tests for continuous and χ^2^-tests for categorical variables.

Intent-to-treat analyses for the effect of inpatient treatment on BMI, BSI depression scores, BSI GSI, GAF, and the EDI-2 mean total score as well as mean scores for the respective subscales in patients with AN and AN+BPD and Group x Time Interactions were conducted with longitudinal multilevel mixed effects models for change, which is considered the method of choice to assess clinical outcomes in longitudinal studies with a high percentage of missing values [24].

Cohen’s *d* effect sizes were calculated for pre- to post-treatment changes for all outcomes, using the estimated marginal means and the standard deviation at pre-treatment of the respective variables. Analyses were performed for the full sample and separately for the two diagnostic groups. Statistical analyses were conducted with SPSS 25 for Macintosh.

## Results

Between January 01, 2013, and December 31, 2017, of a total of 5053 patients treated for an eating disorder in the Schoen Clinic Roseneck, 1160 met the inclusion criteria. Of these patients, 1127 (97.2%) were female, and 68 (5.9%) had a comorbid BPD. At admission, the patients had a mean age of 26.15 years (*SD* = 9.41, range 18–74) and an average BMI of 14.53 kg/m^2^ (*SD* = 1.82, range 9.25–17.50). Table 1 displays the sample descriptive statistics for the two diagnostic groups. There were statistically significant differences in the number of previous inpatient treatments, BMI at admission, medication at discharge, and number of psychiatric comorbidities between patients with AN and AN+BPD (all *p* < .001). Patients with AN+BPD showed statistically significant more previous inpatient treatments and higher rates of at least one additional comorbidity and were more frequently treated with medication at discharge. Differences at admission in the other examined variables are shown in Supplement 2.

**Table 1:** Differences between inpatients with AN and AN+BPD

Variable	AN	AN+BPD	*p*
Age (years); *M* (*SD*)	26.12 (9.55) (*N* = 1092)	26.65 (6.85) (*N* = 68)	.549
Female gender; N (%)	1059 (97.0) (*N* = 1092)	68 (100) (*N* = 68)	.146
No. of previous inpatient treatments; *M SD)*	1.94 (2.97) (*N* = 1054)	3.86 (3.49) (*N* = 65)	<.001
No. of previous psychotherapeutic treatments; *M SD)*	0.67 (0.96) (*N* = 140)	1.44 (1.74) (*N* = 9)	.222
No. of previous psychiatric treatments; *M SD)*	0.17 (0.43) (N = 140)	0.89 (1.69) (N = 9)	.240
Weight in kg at admission; *M* (*SD*)	40.40 (6.36) (N = 1092)	41.88 (5.83) (N = 68)	.061
BMI (kg/m^2^) at admission; M (*SD*)	14.5 (1.82) (N = 1092)	14.99 (1.83) (N = 68)	.030
Weight in kg at discharge; *M* (*SD*)	47.52 (7.33) (*N* = 1001)	48.06 (6.62) (*N* = 63)	.567
BMI (kg/m^2^) at discharge; M (*SD*)	17.09 (2.14) (N = 1001)	17.18 (2.13) (N = 63)	.728
Total weight gain in kg; *M* (*SD*)	7.26 (5.21) (N = 1001)	6.19 (5.87) (N = 63)	.117
Weekly weight gain in kg; *M* (*SD*)	0.61 (0.92) (N = 1001)	0.61 (1.92) (N = 63)	.974
Length of stay (days); *M* (*SD*)	85.33 (49.80) (*N* = 1088)	87.44 (67.14) (*N* = 68)	.741
Ability to work at discharge; N (%)			.756
Fit to work	233 (21.3)	15 (22.1)	
Unfit to work	409 (37.5)	28 (41.2)	
Unclear	450 (41.2)	25 (36.8)	
	(N = 1092)	(N = 68)	
Medication at discharge; N (%)			<.001
Yes	594 (54.4)	57 (83.8)	
No	498 (45.6)	11 (16.2)	
	(N = 1092)	(N = 68)	
Discharge reason; N (%)			.148
Regular	744 (68.1)	34 (50)	
Against medical advice	136 (12.5)	14 (20.6)	
To somatic hospital	42 (3.8)	5 (7.4)	
Other reasons	170 (15.6)	15 (22.0)	
	(N = 1092)	(N = 68)	
No. of psychiatric comorbidities (in addition to BPD in AN+BPD); N (%)			<.001
0	348 (31.9)	8 (11.8)	
≥ 1	744 (68.1)	60 (88.2)	
	(N = 1092)	(N = 68)	

*Note*. *AN* anorexia nervosa, *AN+BPD* anorexia nervosa with comorbid Borderline personality disorder, *BMI* body mass index, *M* mean, *SD* standard deviation, *p* = statistical significance of independent *t*-tests for continuous and χ^2^-tests or Fisher’s exact test for categorical variables (corrected for unequal variances, if required), kg = kilogram

**Fig. 1:**
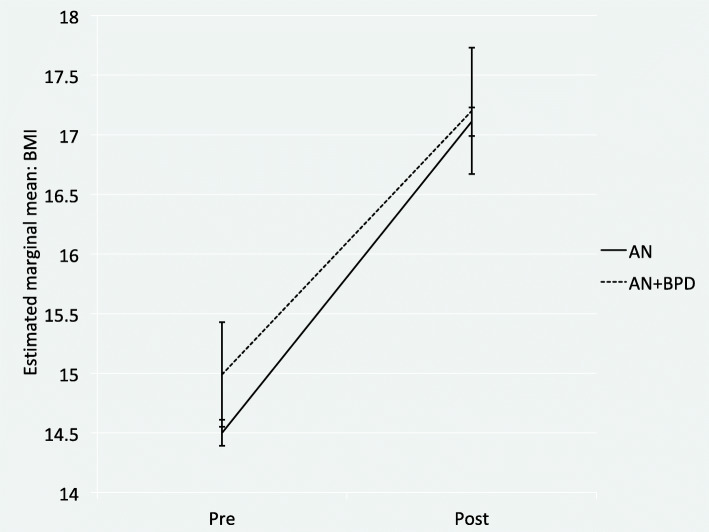
Increase in body mass index (BMI) for inpatients with AN (N = 1092) and AN+BPD (*N* = 86) from pre- to post-treatment. *Note*. Error bars represent 95% confidence intervals. AN = anorexia nervosa, AN+BPD = anorexia nervosa with comorbid Borderline personality disorder

**Fig. 2:**
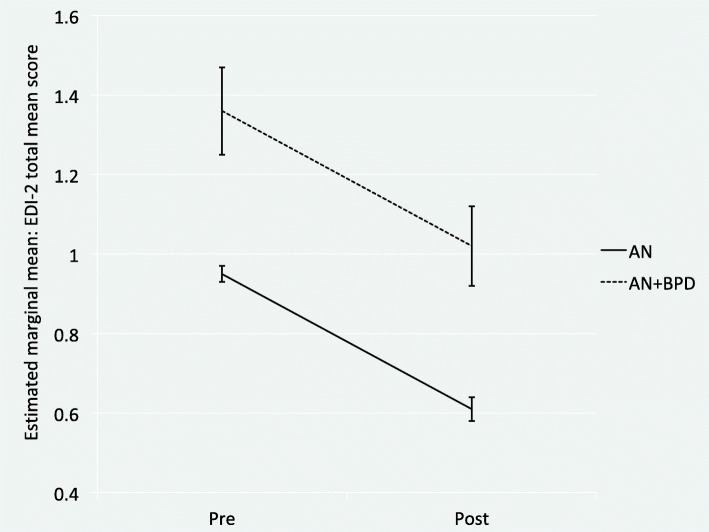
Change in eating disorder symptoms for inpatients with AN (*N* = 882) and AN+BPD (*N* = 53) from pre- to post-treatment. *Note*. Error bars represent 95% confidence intervals. AN = anorexia nervosa, AN+BPD = anorexia nervosa with comorbid Borderline personality disorder, EDI-2 = Eating Disorder Inventory 2

**Fig. 3:**
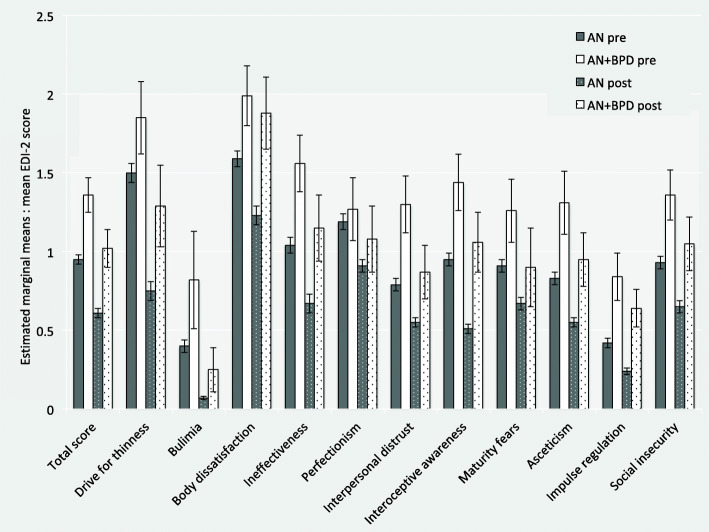
Admission and discharge values of EDI-2 mean scores in inpatients with AN (*N* = 882) and AN+BPD (*N* = 53) from pre- to post-treatment. Error bars represent 95% confidence intervals. AN = anorexia nervosa, AN+BPD = anorexia nervosa with comorbid Borderline personality disorder, EDI-2 = Eating Disorder Inventory 2

Longitudinal multilevel mixed-effects models revealed that the full sample as well as patients with AN statistically significantly improved in all variables (*Tables* *2* and *3*). Patients with AN+BPD significantly improved in about half of the examined variables. Most effect sizes were small to moderate. Improvements with large effects were found for BMI, BSI depression scores, the BSI GSI, and GAF scores in patients with AN. Patients with AN+BPD showed improvements with large effect sizes in BMI, BSI depression scores, the BSI GSI, and the EDI-2 total mean score. Patients with AN showed the smallest gains in EDI-2 perfectionism mean scores, patients with AN+BPD in EDI-2 body dissatisfaction mean scores. Figures 1 and 2 give visualized changes in BMI and EDI-2 total score for the two groups. The inspection of the respective 95% confidence intervals of the estimated marginal means suggests that discharge scores of patients with AN+BPD often lay in the range of admission scores of patients with AN. Figure 3 exemplifies this pattern for the EDI-2 mean scores.

**Table 2:** Effect of treatment on weight, depressive symptoms, general psychopathology, psychosocial functioning, and eating disorder symptoms in the full sample: results of the longitudinal multilevel mixed effects models

Outcome	N	*M* pre (95% CI)	*M* post (95% CI)	*d* (95% CI)	
BMI (kg/m^2^)	1160	14.53 (14.42; 14.63)	17.11 (16.99; 17.24)	1.42 (1.29; 1.55)	
BSI DEP	941	69.72 (69.07; 70.36)	61.29 (60.50; 62.08)	0.83 (0.70; 0.96)	
BSI GSI	941	72.22 (71.56; 72.87)	61.92 (61.04; 62.80)	1.00 (0.86; 1.13)	
GAF	564	39.95 (38.95; 40.95)	53.86 (52.51; 55.22)	1.15 (0.97; 1.33)	
EDI-2 TO	935	0.97 (0.94; 1.00)	0.63 (0.60; 0.66)	0.76 (0.62; 0.89)	
EDI-2 DT	935	1.52 (1.46; 1.58)	0.78 (0.72; 0.83)	0.80 (0.66; 0.93)	
EDI-2 BU	935	0.42 (0.38; 0.47)	0.08 (0.06; 0.10)	0.49 (0.36; 0.62)	
EDI-2 BD	935	1.61 (1.56; 1.66)	1.26 (1.20; 1.32)	0.46 (0.33; 0.59)	
EDI-2 IN	935	1.07 (1.02; 1.11)	0.68 (0.64; 0.73)	0.54 (0.41; 0.67)	
EDI-2 PE	935	1.19 (1.14; 1.24)	0.92 (0.88; 0.97)	0.37 (0.24; 0.49)	
EDI-2 ID	935	0.82 (0.78; 0.86)	0.57 (0.54; 0.60)	0.40 (0.27; 0.53)	
EDI-2 IA	935	0.98 (0.93; 1.02)	0.54 (0.50; 0.58)	0.67 (0.54; 0.80)	
EDI-2 MF	935	0.93 (0.89; 0.97)	0.68 (0.64; 0.72)	0.39 (0.26; 0.51)	
EDI-2 AS	935	0.86 (0.82; 0.90)	0.58 (0.54; 0.61)	0.46 (0.33; 0.59)	
EDI-2 IR	935	0.44 (0.41; 0.47)	0.26 (0.24; 0.29)	0.42 (0.29; 0.55)	
EDI-2 SI	935	0.95 (0.92; 0.99)	0.67 (0.63; 0.70)	0.48 (0.35; 0.61)	

*Note*. All pre to post changes are significant with *p* < 0.001. *AN* anorexia nervosa, *AN+BPD* anorexia nervosa with comorbid Borderline personality disorder, *M* = estimated marginal mean, *SE* standard error, *d* = Cohen’s *d* (calculated with the standard deviation of the pre-treatment values), *BMI* body mass index, BSI DEP = T-score for the depression scale of the Brief Symptom Inventory, *BSI GSI* = T-score for the global severity index of the Brief Symptom Inventory, *GAF* Global Assessment of Functioning. Abbreviations for the Eating Disorder Inventory 2 (EDI-2) scales: *TO* total, *DT* drive for thinness, *BU* bulimia, *BD* body dissatisfaction, *IN* ineffectiveness, *PE* perfectionism, *ID* interpersonal distrust, *IA* interoceptive awareness, *MF* maturity fears, *AS* asceticism, *IR* impulse regulation, *SI* social insecurity

**Table 3:** Effect of treatment on weight, depressive symptoms, general psychopathology, psychosocial functioning, and eating disorder symptoms in inpatients with AN and AN+BPD: results of the longitudinal multilevel mixed effects models

Outcome		AN					AN+BPD			
	N	*M* pre (95% CI)	*M* post (95% CI)	*p (within-group)*	*d* (95% CI)	N	*M* pre (95% CI)	*M* post (95% CI)	*p (within-group*	*d* (95% CI)
BMI	1092	14.50 (14.39; 14.61)	17.09 (16.95; 17. 22)	<.001	1.40 (1.31; 1.48)	68	14.99 (14.56; 15.42)	17.18 (16.66; 17.70)	<.001	1.08 (0.77; 1.39)
BSI DEP	887	69.27 (68.60; 69.94)	60.73 (59.90; 61.56)	<.001	0.79 (0.70; 0.87)	54	75.50 (73.87; 77.13)	67.91 (64.61; 71.21)	<.001	0.69 (0.33; 1.03)
**BSI GSI**	**887**	**71.87 (71.18; 72.56)**	**61.23 (60.30; 62.16)**	**<.001**	**0.95 (0.86; 1.03)**	**54**	**77.24 (75.72; 78.76)**	**70.64 (67.43; 73.85)**	**<.001**	**0.65 (0.30; 0.99)**
**GAF**	**530**	**40.12 (39.09; 41.15)**	**54.46 (53.07; 55.85)**	**<.001**	**0.98 (0.87; 1.08)**	**34**	**37.32 (33.35; 41.29)**	**44.53 (39.47; 49.59)**	**.008**	**0.48 (0.13; 0.84)**
EDI-2 TO	882	0.94 (0.91; 0.97)	0.60 (0.57; 0.63)	<.001	0.96 (0.87; 1.04)	53	1.35 (1.24; 1.46)	1.01 (0.88; 1.14)	<.001	0.99 (0.59; 1.37)
EDI-2 DT	882	1.49 (1.43; 1.55)	0.74 (0.68; 0.80)	<.001	0.95 (0.86; 1.04)	53	1.82 (1.60; 2.04)	1.33 (1.07; 1.59)	<.001	0.80 (0.43; 1.16)
EDI-2 BU	882	0.40 (0.36; 0.44)	0.07 (0.05; 0.09)	<.001	0.51 (0.43; 0.58)	53	0.73 (0.49; 0.97)	0.23 (0.08; 0.38)	<.001	0.71 (0.35; 1.07)
**EDI-2 BD**	**881**	**1.58 (1.53; 1.63)**	**1.23 (1.17; 1.29)**	**<.001**	**0.53 (0.45; 0.60)**	**53**	**1.98 (1.80; 2.19)**	**1.89 (1.65; 2.11)**	**.161**	**0.25 (−0.08; 0.57)**
EDI-2 IN	882	1.04 (0.99; 1.09)	0.64 (0.60; 0.68)	<.001	0.66 (0.58; 0.74)	53	1.56 (1.41; 1.77)	1.11 (0.88; 1.34)	<.001	0.77 (0.41; 1.13)
EDI-2 PE	882	1.19 (1.14; 1.24)	0.91 (0.86; 0.96)	<.001	0.50 (0.43; 0.58)	53	1.24 (1.04; 1.44)	1.07 (0.84; 1.30)	.027	0.37 (0.04; 0.70)
**EDI-2 ID**	**882**	**0.79 (0.75; 0.83)**	**0.54 (0.50; 0.58)**	**<.001**	**0.47 (0.39; 0.54)**	**53**	**1.31 (1.13; 1.49)**	**0.85 (0.67; 1.03)**	**<.001**	**0.84 (0.47; 1.21)**
EDI-2 IA	882	0.94 (0.90; 0.98)	0.51 (0.47; 0.55)	<.001	0.74 (0.66; 0.82)	53	1.42 (1.25; 1.59)	1.09 (0.90; 1.28)	.001	0.64 (0.29; 0.99)
EDI-2 MF	882	0.91 (0.87; 0.95)	0.66 (0.62; 0.70)	<.001	0.46 (0.39; 0.54)	53	1.26 (1.07; 1.45)	0.82 (0.67; 0.97)	<.001	0.48 (0.14; 0.81)
EDI-2 AS	880	0.83 (0.79; 0.87)	0.54 (0.50; 0.58)	<.001	0.56 (0.48; 0.64)	53	1.30 (1.11; 1.49)	0.96 (0.78; 1.14)	<.001	0.64 (0.29; 0.99)
EDI-2 IR	880	0.41 (0.38; 0.44)	0.24 (0.22; 0.26)	<.001	0.48 (0.40; 0.55)	53	0.83 (0.69; 0.97)	0.63 (0.50; 0.76)	.003	0.46 (0.12; 0.79)
EDI-2 SI	880	0.93 (0.89; 0.97)	0.64 (0.60; 0.68)	<.001	0.60 (0.52; 0.68)	53	1.36 (1.20; 1.52)	1.00 (0.82; 1.18)	<.001	0.62 (0.02; 1.07)

*Note*. Significant Group x Time Interactions are **boldface**. *AN* anorexia nervosa, *AN+BPD* anorexia nervosa with comorbid Borderline personality disorder, *M* = estimated marginal mean, *95% CI* 95% confidence interval, *d* = Cohen’s *d* (calculated with the standard deviation of the pre-treatment values), *BMI* body mass index, BSI DEP = T-score for the depression scale of the Brief Symptom Inventory, BSI GSI = T-score for the global severity index of the Brief Symptom Inventory, *GAF* Global Assessment of Functioning. Abbreviations for the Eating Disorder Inventory 2 (EDI-2) scales: *TO* total, *DT* drive for thinness, *BU* bulimia, *BD* body dissatisfaction, *IN* ineffectiveness, *PE* perfectionism, *ID* interpersonal distrust, *IA* interoceptive awareness, *MF* maturity fears, *AS* asceticism, *IR* impulse regulation, *SI* social insecurity

## Discussion

In a large sample of inpatients with AN and AN+BPD, we found with regard to our study aims: (1) There were no differences in admission and discharge characteristics, including demographic variables, weight, rate of weight gain, and ability to work between inpatients with AN and AN+BPD, except previous inpatient treatments, BMI at admission medication at discharge and the frequency of at least one comorbidity, with higher values for AN+BPD. (2) Patients with AN+BPD presented to treatment with higher symptom loads across all measures. Even though higher treatment gains in half of the variables for patients with AN+BPD, their discharge values were in the range of or even higher than scores at admission for AN. Thereby, our findings extend those of preliminary studies suggesting aggravated ED symptoms and less response to treatment in patients with AN and comorbid BPD [7, 15, 16] or other PDs [4, 12–14] and parallel similar studies with patients with BN and comorbid PDs [7–11].

With a rate of 5.9%, BPD was less frequent in our sample than the 19% suggested by meta-analyses [1]. Around half of the variation in the prevalence of comorbid PDs in eating disorders is assumed to result from the setting and depends on used assessment methods (e.g., structured PD diagnostics vs. PD diagnostics using unstructured clinical interviews) [1]. Lower rates are usually found for clinical-unstructured assessments [25]. The low prevalence of BPD we found in our study in an inpatient setting might be in part explained by the fact that conducted standard clinical interviews at admission were not complemented by standardized personality disorder diagnostics. Also, a selection bias might have occurred. First, while being specialized in the treatment of eating disorders, our clinic does not offer a BPD-specific program. As a result, many patients with BPD may have opted to seek treatment in BPD-specialized clinics. Second, either the patients with AN+BPD themselves or the referring physicians erroneously assumed that a psychiatric setting focusing on pharmacological treatment would be more appropriate given the severity of the disorders. As our clinic emphasizes psychotherapy with adjuvant pharmacological treatment, fewer patients with AN+BPD would have sought treatment here.

Contrary to other studies [7, 13, 26], we found in total no significant differences in the distribution of discharge reasons (e.g., regular or against medical advice) for comorbid patients compared to AN in our sample. This result might be explained by the multimodal treatment, offering the patients a range of therapeutic approaches and contact persons, especially the co-therapists as close contact persons to the patients during daily routines on the ward. However, frequency of discharge against medical advice was higher in AN+BPD than for AN in absolute terms, even if not significantly different, which can be interpreted as a higher dropout rate from inpatient treatment in AN+BPD.

Even though there were no differences between groups in the ability to work at discharge, the lower GAF ratings in the comorbid group, indicating severe symptoms or serious psychosocial impairment even after treatment, are in line with a previous study [7]. While non-comorbid patients gained slightly more weight during treatment, the comorbid group presented to treatment with half a BMI point more on average, representing a significant group difference at admission. A similar pattern was found in a study examining the influence of comorbid PDs on weight gain during inpatient treatment for AN [13].

Patients with AN+BPD presented with worse depressive symptoms, general psychopathology, and all EDI-2 mean scores except for perfectionism. Comparing the respective pre-post effect sizes between the diagnostic groups suggests that comorbid patients were able to achieve larger gains in the EDI-2 total mean score as well as bulimia, ineffectiveness, interpersonal distrust, maturity fears, asceticism, and social insecurity subscores, with confidence intervals for the effect sizes, however, overlapping. Smaller effect sizes were observed for comorbid patients in depressive symptoms, general psychopathology, and EDI-2 drive to thinness, body dissatisfaction, perfectionism, interoceptive awareness, and impulse regulation. Again, results revealed overlapping confidence intervals for the effect sizes.

Notably, most discharge scores for the comorbid group were as high or even higher than the admission scores of the non-comorbid group. In patients with AN+BPD, the highest scores at admission in EDI-2 were found for body dissatisfaction, drive for thinness, ineffectiveness, and interoceptive awareness. Treatment gains for comorbid patients were the smallest for impulse regulation, body dissatisfaction, and perfectionism. Group x Time Interactions revealed significant group differences with reduced treatment benefits in AN+BPD for general psychopathology (BSI-GSI, GAF) and EDI-2 body dissatisfaction as specific eating disorder psychopathology. However, patients with AN+BPD showed elevated treatment response in EDI-2 interpersonal distrust compared to patients with AN.

### Clinical implications

Our findings emphasize that persons with AN+BPD present to inpatient treatment with a range of severe symptoms and pronounced psychosocial impairment. While they seem to benefit from multimodal specialized inpatients treatment and patterns of weight gain did not deviate from patients with AN only, these patients often retain substantial levels of psychopathology and impairment at discharge. In particular, both core symptoms of eating disorders like body dissatisfaction, drive for thinness, and perfectionism, as well as core competencies of emotion regulation, including impulse regulation and interoceptive awareness, need to be even more addressed in the treatment of these patients. These findings are in line with two trials reporting only small improvements in patients with comorbid eating disorders and BPD, even under treatment with BPD-specific elements [7, 8]. Given the same average treatment duration for patients with AN and AN+BPD in our study, it remains to be investigated whether the comorbid patients would have benefitted from prolonged treatment. However, a similar investigation from our clinic examined the influence of BPD on inpatient treatment for bulimia nervosa found comparable patterns of symptom severity and outcome despite prolonged treatment for comorbid patients [9].

It is likely that change occurs more slowly for patients with two mental disorders as severe as AN and BPD and affords repeated treatment efforts across the lifespan. Nevertheless, existing treatment options need to be improved. Approaches incorporating the functional associations between individual symptoms of the disorders might help to do so both conceptually and pragmatically. The individual patient’s symptomatology could be categorized in the sense of disorder-specific symptoms of AN or BPD and an area of overlap, containing transdiagnostic or “bridging” symptoms shared by both AN and BPD. Preliminary evidence for this notion is provided by a network analysis of eating disorder and BPD symptoms in a community sample, suggesting that disorder-specific symptoms are linked by emotion dysregulation and abandonment as central variables of the network [27]. An accurate and early diagnosis of relevant personality traits is crucial for this approach. Attention to the AN-subtype might further improve the understanding of how personality and eating disorder symptoms relate. Avoidant, dependent, and obsessive-compulsive personality traits seem to be more common in restrictive AN and Borderline traits to be more prevalent in binge/purge AN [28]. Given the multitude of Borderline syndromes resulting from the 5 out of 9 symptoms rule, it has been argued that only specific aspects of BPD are associated with core symptoms of AN [29]. While interpersonal and emotional problems are similarly related to both AN and BN, impulsivity is related exclusively to BN and identity disturbance overly to AN. Difficulties in emotion regulation, however, are shared by all PDs and AN [30]. PD symptoms have accordingly been linked with emotion regulation deficits and not eating disorders symptoms in AN [31].

### Strengths and limitations

Major strengths of our study are the large sample size, the longitudinal design, and the high external validity, which did not pertain to any of the few previous studies in this area. Limitations include the lack of a follow-up examination after discharge, the unavailability of data on the AN-subtype, and that BPD was not diagnosed by a standardized interview. However, results in the literature indicate sufficient diagnostic agreement between standardized diagnostic interviews and clinical diagnoses both for AN and BPD if given by highly experienced and trained clinicians as in our case [32–34]. Due to a lack of sufficient knowledge about the differential influence of comorbid BPD on treatment outcome in AN, we would like to note that conducted analyses are in a part of exploratory nature.

## Conclusions

Patients with AN+BPD show higher levels of disorder-specific and general psychopathology (except perfectionism) at admission, substantial psychosocial impairment in daily functioning, and a higher, although not significantly different, rate of dropping out from treatment. Even though these patients retained high levels of impairment at discharge, they were able to benefit from specialized multimodal inpatient treatment. There is tentative evidence for partially reduced treatment response in comorbid patients with AN+BPD compared to patients with AN, especially for general psychopathology and specific facets like impulse regulation, perfectionism, or body dissatisfaction. Treatment for this vulnerable group might improve through targeting transdiagnostic symptoms central to both disorders, especially emotion dysregulation. Prospectively, further studies in this understudied subject are needed to identify other domains of reduced treatment response in AN+BPD and foster the development and application of more targeted interventions for these crucial areas of treatment effectiveness.

## Supplementary Information

**Additional file 1.** Supplement 1: Excerpt of the recommendations for inpatient treatment of anorexia. Nervosa according to German S3-guidelines.

**Additional file 2.** SUPPLEMENT 2: Differences between inpatients with AN and AN+BPD at admission.

## Data Availability

The dataset supporting the conclusion of this article are held by the authors and will be made available upon justified request.

## Abbreviations

AN: Anorexia nervosa
AN+BPD: Comorbid anorexia nervosa and Borderline personality disorder
BMI: Body mass index
BPD: Borderline personality disorder
BSI: Brief Symptom Inventory
EDI-2: Eating Disorder Inventory 2
GAF: Global Assessment of Functioning
GSI: Global severity index of the Brief Symptom Inventory
SD: Standard deviation
