# Supplementary material for: Impact of comorbid borderline personality disorder on the outcome of inpatient treatment for anorexia nervosa: a retrospective chart review
Source: Borderline Personal Disord Emot Dysregul. 2021 Mar 11;8:8. doi: 10.1186/s40479-021-00149-7 (PMC7948359; doi:10.1186/s40479-021-00149-7)
Supplement: Supplementary file 1 — Additional file 1. Supplement 1: Excerpt of the recommendations for inpatient treatment of anorexia. Nervosa according to German S3-guidelines. [file 40479_2021_149_MOESM1_ESM.pdf]

Supplement 1: Excerpt of the recommendations for inpatient treatment of anorexia nervosa according to German S3-guidelines

(Deutsche Gesellschaft für Essstörungen (2019). *Gemeinsame S3-Leitlinie „Diagnostik und Therapie der Essstörungen“*. AWMF.)

- Patients should receive inpatient treatment if one or more of the following criteria are fulfilled:
  - Rapid or continued weight loss (>20% in the past six months)
  - Extremely low body weight (BMI <15 kg/m² or <3rd age- and sex-specific percentile for children and adults)
  - Continued weight loss or insufficient weight gain in three months despite outpatient or daypatient treatment
  - Social/family factors that interfere with the recovery process
  - Severe comorbid mental disorders
  - Suicidality
  - Severe bulimic symptomatology and/or excessive compulsive exercise that cannot be treated with outpatient therapy
  - Compromised physical health or medical complications
  - Low insight into illness
  - Overload in outpatient treatment setting
  - Need for treatment by a multiprofessional team with typical hospital-based methods

- Patients should receive inpatient treatment in an institution that offers a specialized, multimodal treatment program.

- In addition to the standard treatment elements of a psychosomatic-psychotherapeutic inpatient treatment or a psychiatric-psychosomatic inpatient treatment for children and adolescents, the following disorder-specific therapy elements should be included in the specialized therapy program:
    - ➢ Disorder-oriented psychotherapy (individual and group setting)
    - ➢ Disorder-oriented nutritional management
    - ➢ Interventions that aim at a modification of body weight-related, eating-related, and (if necessary) exercise-related behaviors
    - ➢ Medical treatment and regular rounds with discussion of the weight trajectory
    - ➢ Disorder-oriented body image-related and (if necessary) exercise therapy
    - ➢ Involvement of the family (at least for children and adolescents)

- The inpatient treatment should aim at a restoration of body weight (BMI >18.5 kg/m² or 25th age- and sex-specific percentile for children and adolescents, at least 10th percentile); however, an individual target weight can be determined taking other factors into account.

- The inpatient treatment should aim at a weekly body weight gain of 500–1000 g.

- Patients should be weighed regularly (1–3 times per week) with an empty stomach in the morning at approximately the same time in light clothing.

- To reduce the risk of relapse, the final stage of inpatient treatment should aim at weight maintenance after discharge and prepare outpatient aftercare.

- Because of high risk of relapse, the transition between treatment settings should receive special attention and linkage to outpatient psychotherapists should be sought actively; support groups or online therapies can be used to facilitate the transition to the outpatient treatment setting; (re-)integration into school or occupational environment should also be part of the transition preparation.

- The inpatient treatment should be followed by outpatient psychotherapy; for children and adolescents, this outpatient psychotherapy should be family-based psychotherapy (unless there are serious contraindications).
