# Supplementary material for: Impact of comorbid borderline personality disorder on the outcome of inpatient treatment for anorexia nervosa: a retrospective chart review
Source: Borderline Personal Disord Emot Dysregul. 2021 Mar 11;8:8. doi: 10.1186/s40479-021-00149-7 (PMC7948359; doi:10.1186/s40479-021-00149-7)
Supplement: Supplementary file 2 — Additional file 2. SUPPLEMENT 2: Differences between inpatients with AN and AN+BPD at admission. [file 40479_2021_149_MOESM2_ESM.pdf]

SUPPLEMENT 2: Differences between inpatients with AN and AN+BPD at admission.

Variable M(SD)	AN (N=1092)	AN + BP (N=68)	*p*
BSI DEP	69.27 (10.24)	75.5 (6.1)	<.001
BSI GSI	71.87 (10.48)	77.24 (5.7)	<.001
GAF (2 months prior admission)	40.12 (12.09)	37.32 (11.8)	.192
EDI-2 TO	0.94 (0.44)	1.35 (0.41)	<.001
EDI-2 DT	1.49 (0.93)	1.82 (0.82)	.007
EDI-2 BU	0.40 (0.68)	0.73 (0.88)	.009
EDI-2 BD	1.58 (0.76)	1.98 (0.73)	<.001
EDI-2 IN	1.03 (0.72)	1.56 (0.68)	<.001
EDI-2 PE	1.19 (0.74)	1.24 (0.75)	.629
EDI-2 ID	0.79 (0.62)	1.31 (0.66)	<.001
EDI-2 IA	0.94 (0.65)	1.42 (0.65)	<.001
EDI-2 MF	0.90 (0.64)	1.25 (0.72)	<.001
EDI-2 AS	0.83 (0.52)	1.3 (0.72)	<.001
EDI-2 IR	0.41 (0.42)	0.83 (0.52)	<.001
EDI-2 SI	0.93 (0.58)	1.36 (0.59)	<.001
incapacity to work at admission (weeks)	14.08 (23.92)	25.93 (34.4)	.035

Note. AN = anorexia nervosa, AN+BPD = anorexia nervosa with comorbid Borderline personality disorder, *M* = estimated marginal mean, *SD* = standard deviation, BSI DEP = T-score for the depression scale of the Brief Symptom Inventory, *p* = statistical significance of independent *t*-tests for continuous variables, BSI GSI = T-score for the global severity index of the Brief Symptom Inventory, GAF = Global Assessment of Functioning. Abbreviations for the Eating Disorder Inventory 2 (EDI-2) scales: TO = total, DT = drive for thinness, BU = bulimia, BD = body dissatisfaction, IN = ineffectiveness, PE = perfectionism, ID = interpersonal distrust, IA = interoceptive awareness, MF = maturity fears, AS = asceticism, IR = impulse regulation, SI = social insecurity.
